# Perspectives on youth‐onset nonalcoholic fatty liver disease

**DOI:** 10.1002/edm2.184

**Published:** 2020-09-17

**Authors:** Eduardo Castillo‐Leon, Catherine E. Cioffi, Miriam B. Vos

**Affiliations:** ^1^ Department of Pediatrics Emory University School of Medicine Atlanta GA USA; ^2^ Nutrition & Health Sciences Doctoral Program Laney Graduate School Emory University Atlanta GA USA; ^3^ Children's Healthcare of Atlanta Atlanta GA USA

**Keywords:** children, nonalcoholic fatty liver disease, type 2 diabetes

## Abstract

**Background:**

The prevalence and incidence of youth‐onset nonalcoholic fatty liver disease (NAFLD) far exceeds other paediatric chronic liver diseases and represents a considerable public health issue globally.

**Methods:**

Here, we performed a narrative review of current knowledge regarding the epidemiology of paediatric NAFLD, selected concepts in pathogenesis, comorbidities, diagnosis, and management, and issues related to the transition to adulthood.

**Results:**

Paediatric NAFLD has become increasingly more prevalent, especially in certain subgroups, such as children with obesity and certain races/ethnicities. The pathophysiology of paediatric NAFLD is complex and multifactorial, driven by an interaction of environmental and genetic factors. Once developed, NAFLD in childhood is associated with type 2 diabetes, hypertension, increased cardiovascular disease risk, and end‐stage liver disease. This predicts an increased burden of morbidity and mortality in adolescents and young adults. Early screening and diagnosis are therefore crucial, and the development of noninvasive biomarkers remains an active area of investigation. Currently, treatment strategies are focused on lifestyle changes, but there is also research interest in pharmacological and surgical options. In the transition from paediatric to adult care, there are several potential challenges/barriers to treatment and research is needed to understand how best to support patients during this time.

**Conclusions:**

Our understanding of the epidemiology and pathophysiology of paediatric NAFLD has increased considerably over recent decades, but several critical knowledge gaps remain and must be addressed in order to better mitigate the short‐term and long‐term risks of youth‐onset NAFLD.

## Graphical abstract

The prevalence and incidence of youth‐onset nonalcoholic fatty liver disease (NAFLD) far surpasses other chronic liver diseases. In children, NAFLD is associated with type 2 diabetes, hypertension, increased cardiovascular disease risk, and end‐stage liver disease. This predicts an increased burden of morbidity and mortality in adolescents and young adults.

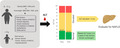

## 1 INTRODUCTION AND THE GROWING PREVALENCE OF NAFLD

Nonalcoholic fatty liver disease (NAFLD) is becoming increasingly well‐known for being the most common chronic liver disease in the world. While NAFLD has been documented across the full lifespan, from newborn to those advanced in age, the increase in children with NAFLD is of particular concern. Prior studies suggest that youth with NAFLD exhibit a more progressive form of the disease, including increased fibrosis, compared to adults with NAFLD.^1^ Further, the health consequences associated with early‐onset chronic liver disease in children are likely severe and long term. For example, the increasing number of liver transplant registrants among younger adults (35‐55 years) is likely explained by increased youth‐onset of NAFLD.^2^ The rise of paediatric NAFLD parallels the increase in obesity in children, a leading risk factor for NAFLD^3^; however, it is also true that not all children with obesity develop NAFLD and a gene‐environment interaction likely underlies disease susceptibility as well. In this review, we discuss the current understanding of the epidemiology of youth‐onset NAFLD, selected concepts in pathogenesis relevant to the childhood form of NAFLD, comorbidities, diagnosis and management, and issues related to the transition to adulthood for children with NAFLD.

## 2 EPIDEMIOLOGY

Most epidemiological studies thus far have relied on alanine aminotransferase (ALT) or imaging such as ultrasound or magnetic resonance imaging (MRI) to estimate the prevalence of NAFLD. Although the prevalence of NAFLD varies due to the sensitivity of the diagnostic method used, multiple studies describe a higher prevalence of NAFLD in children with obesity.^4^, ^5^ Notably, in a recent meta‐analysis by Anderson et al, it was estimated that the global prevalence of paediatric NAFLD is 7.6% (95% confidence interval [CI]: 5.5%‐10.3%) in the general population, but as high as 34.2% (95% CI: 27.8%‐41.2%) in children with obesity.^5^ Prevalence also varied according to geographic region. Specifically, in general population studies, the estimated prevalence was highest in South America, at 25.1%, whereas the other regions were similar (ranging 5.7%‐10.0%), and in clinical population studies, the prevalence was highest in Asia at 62.3%.^5^ On average, there was also a higher prevalence among males than females.^5^

Although this meta‐analysis had a large between‐study heterogeneity (*i* ^2^ = 98%) likely due to differences in sample size and diagnostic modality, a few more recent studies also reported a high prevalence of NAFLD among children with obesity. One published in 2019 by Yu et al^6^ examined the prevalence of NAFLD is a cohort of 408 children (9‐17 years) with obesity and found that 29% of males and 23% of females were diagnosed with NAFLD based on MRI‐proton density fat fraction. This prevalence was adjusted for race/ethnicity due to high proportion of Hispanic children in the cohort (77%). In a more representative study by Tricò et al^7^ of 503 adolescents (38% White, 26% Black and 35% Hispanic) recruited from an obesity clinic, the prevalence of NAFLD was 41% overall, but varied by race/ethnicity with the highest prevalence among Hispanic youth (60%), followed by white (43%) and black children (16%).

Other studies have also confirmed that the prevalence of NAFLD varies within the US according to race/ethnicity. For instance, in two population‐based autopsy studies conducted in New York and California, the overall prevalence of paediatric NAFLD differed considerably at 4.5% and 13%, respectively,^8^, ^9^ a difference that was mainly driven by the race/ethnicity distribution in each study. Specifically, the New York cohort had a higher percentage of black children (50% of the sample), among whom there was a very low prevalence of NAFLD of 1%. It is important to emphasize, however, that in the study by Tricò et al^7^ from Connecticut, 16% of black children presented with NAFLD by MRI. These higher estimates are likely explained by the overall higher adiposity of the Connecticut cohort (mean BMI *z*‐score 2.2 ± 0.4) compared to the New York cohort (mean BMI *Z*‐score: 0.63 ± 1.27), suggesting that black children are still susceptible, but at a higher BMIs.

In the future, genetic testing for NAFLD may better predict risk of disease compared to the current generalizations by race and ethnicity. In addition, it is important to point out that much less is known regarding the population prevalence of steatohepatitis or different fibrosis stages in childhood and adolescence because these require a liver biopsy to detect. Because there is strong interest in developing new technologies for noninvasively assessing these disease end‐points, there will likely be an increase in publications on this topic in the near future.

## 3 NATURAL HISTORY

Overall, there are still many questions with regard to the natural history of NAFLD, especially in children, as most studies to date have been conducted in adults. It appears, however, that most cases of cirrhosis in young adults are a consequence of an undiagnosed youth‐onset of NAFLD. A population‐based cohort study conducted in Canada showed that Millennials (born in 1980 or later) present with the highest incidence of cirrhosis compared to previous generations, and that in this generation NAFLD is responsible for 57% of cirrhosis cases.^10^ Thus, understanding the natural history of NAFLD more holistically, from conception to adulthood, is essential in order to better prevent and/or manage the disease.

Perinatal factors may influence the development of NAFLD during childhood.^11^, ^12^ Maternal factors such as pre‐pregnancy obesity,^13^ gestational diabetes^14^ and excess weight gain during pregnancy^15^ have shown to be strongly associated with hepatic fat in the offspring as early as the neonatal period. Children with low and high birth weights are more likely to progress to more severe stages of fibrosis and steatosis later in adolescence, suggesting a very early influence of nutritional status on future disease progression.^16^ In one study, breastfeeding exclusively for at least 6 months decreased the odds of developing NAFLD during adolescence.^11^ In addition, animal studies have shown that early postnatal exposure to a high‐fat diet increased the susceptibility to NAFLD through the upregulation of hepatic lipogenesis.^17^

Most children who develop NAFLD are diagnosed between 10 and 13 years old.^18^, ^19^ At presentation, 10%‐25% of them have advanced fibrosis and 20%‐50% have nonalcoholic steatohepatitis (NASH).^20^ However, due to the paucity of longitudinal studies, it remains unclear the average duration of the disease prior to diagnosis, as well as the prognosis and long‐term outcomes after diagnosis. There are, however, several smaller long‐term studies that have attempted to describe the natural history of paediatric NAFLD after diagnosis. In a retrospective long‐term follow‐up study of 66 children with NAFLD with a mean follow‐up of 6.4 years, 4 out of 5 children who had a follow‐up biopsies showed progression to a more advanced fibrosis stage.^21^ In another study of 18 children with follow‐up biopsies, 7 had progression of fibrosis stage, and 8 had no change.^22^ Likewise, another retrospective study of 44 children with NAFLD showed that at diagnosis 61% had NASH and 56% had fibrosis based on liver biopsy, and over an average follow‐up of 4.5 years, 30% developed type 2 diabetes.^23^

These studies are biased by the clinical selection of children who are biopsied for a second time, but do highlight the severity of disease seen in some children. This is concerning for several reasons including the observation that NAFLD in childhood tends to persist into adulthood, similar to obesity. In an MRI‐based longitudinal study of 57 adolescents with NAFLD with an average follow‐up of 2.3 years, only 23% resolved their NAFLD with standard of care treatment.^7^ Importantly, small increases or decreases in BMI were significantly associated with progression or resolution of NAFL at follow‐up.^7^ In addition, BMI in late adolescence was reported as a strong and independent predictor of severe liver disease later in life.^24^

It is important to also note that most children are diagnosed with NAFLD during or after puberty, a development period characterized by a physiologically normal state of insulin resistance (IR) that peaks in Tanner stage 2 and promotes fat accretion for acquisition of the adult phenotype.^25^ The interaction between sex hormones, IR, adipose re‐distribution and cytokine/adipokine profiles could all contribute to the gender differences seen in NAFLD through puberty and adulthood with males exhibiting a higher susceptibility than females.^26^ There may also be important differences in disease pathogenesis in children who develop NAFLD at very young ages, for example prior puberty. In a study of 186 children with biopsy‐proven NAFLD, prepubertal children had more severe steatosis, fibrosis and portal inflammation than pubertal and postpubescent patients.^27^ Pathological characteristics of those children in puberty and postpuberty were more similar to NAFLD pattern in adults compared to the early puberty children who are more likely to have a portal pattern of inflammation and steatosis (the ‘paediatric’ pattern).^27^ Additional longitudinal studies are required that aim to better understand the pathogenesis of these different histological patterns, and to describe the transition of youth‐onset NAFLD to adulthood and the impact on hepatic and extra‐hepatic clinical outcomes.

## 4 PATHOGENESIS

Hepatic fat (steatosis), the hallmark feature of NAFLD, occurs due to imbalances in hepatic lipid metabolism, and when free fatty acid (FFA) flux to the liver and/or de novo lipogenesis (DNL) exceeds the liver's ability to oxidize and/or secrete excess lipids as very‐low‐density lipoproteins (VLDLs). This metabolic dysregulation is driven by a complex interaction of genetic and environmental factors and a combination of lipotoxic and glucotoxic mechanisms, as reviewed elsewhere,^28^, ^29^ which over time can progress in severity to steatosis with inflammation, cell injury, and/or fibrosis.

Obesity in particular is a strong and independent risk factor for NAFLD in children and adults and is considered a chronic and systemic inflammatory disease,^30^ which affects multiple organs including the pancreas, muscle, liver and adipose tissue.^31^, ^32^, ^33^ In adipose tissue, inflammation drives an adipokine imbalance, characterized by suppression of adiponectin,^34^ which is positively associated with insulin sensitivity and has been shown to be low in children with NAFLD.^35^ This is critical because IR is universal in children with NAFLD, and likely interacts with NAFLD through a bi‐directional relationship, whereby IR drives FFA flux from adipose tissue, while hepatic fat‐related increases in DNL and beta‐oxidation drive lipid peroxidation, endoplasmic reticulum (ER) stress, and generation of reactive oxygen species. Together, this exacerbates IR and also contributes to oxidative stress‐related liver injury.^36^

Among the different process involved in NAFLD pathogenesis, DNL has been shown to be substantially increased in patients with NAFLD,^37^, ^38^ and is the major contributor of intrahepatic triglycerides (nearly 40% of plasma triglyceride‐rich lipoprotein triglycerides in adults with NAFLD and obesity).^39^ This role of DNL in the pathogenesis NAFLD may have increased relevance for children, who tend to consume more fructose compared to adults,^40^ and since fructose has been shown to induce DNL in humans.^41^ This is because fructose metabolism in the liver occurs largely unregulated and may contribute to upregulation in DNL directly via substrates produced by fructose metabolism, or indirectly via upregulation of lipogenic genes.^42^ Fructose also has been shown to upregulate GLP‐1^43^ and is associated with central adiposity^44^ and increased triglyceride levels in children,^45^ which are all mechanisms related to NAFLD.

The microbiome may also be a key driver of youth‐onset NAFLD, given its ability to influence nutrient utilization, immune function and host gene expression.^46^, ^47^, ^48^ Indeed, despite inter‐individual variability, certain microbial patterns have been associated with paediatric NAFLD. In a study of 124 obese children with or without biopsy‐proven NAFLD, children with NAFLD had lower alpha diversity, which was also correlated with NAFLD severity.^49^ Further, it's proposed that certain dietary patterns, such as high‐fructose^50^ or high‐fat diets,^51^ may cause NAFLD in‐part by promoting dysbiosis and intestinal permeability, leading to the translocation of bacterial products that have been linked with NAFLD and NASH.^51^ Consistent with this, in a small (n = 15) study of adolescents with and without NAFLD, those with NAFLD exhibited higher postprandial endotoxin levels during a fructose feeding challenge—a response that correlated with IR and inflammatory cytokines.^52^ Related to this, bile acid (BA) metabolism, which is intertwined in the gut‐liver axis, has also emerged as an important biological pathway in NAFLD. This is largely due to evidence that BAs can act as key signalling molecules that regulate glucose and lipid metabolism via receptors such as the farnesoid X receptor (FXR) and may therefore influence NAFLD at multiple levels, as reviewed in more detail elsewhere.^53^

## 5 GENETICS

One of the strongest and most studied genetic risk factors for NAFLD worldwide is the single nucleotide polymorphism (SNP) rs738409 C > G in *PNPLA3*, which encodes the protein variant I148M, and has been associated with hepatic steatosis and fibrosis progression in children and adults.^54^, ^55^ *PNPLA3* I148M induces hepatic steatosis by retaining polyunsaturated triglycerides, and increasing lipogenesis,^56^ and in children, evidence suggests that it may be a stronger predictor than IR and BMI.^57^ Other genetic variants associated with paediatric NAFLD risk include SNPs in the Transmembrane 6 Superfamily Member 2 (*TM6SF2*) gene,^58^ which results in impaired lipid mobilization via very‐low‐density lipoprotein (VLDL)^59^; and the glucokinase regulatory protein (*GCKR*) gene,^60^ which results in the activation hepatic glucose uptake, blocks fatty acid oxidation, and promotes lipogenesis.^61^ Interestingly, as shown by Goffredo et al, a combined risk score including the *PNPLA3* rs738409, *GCKR* rs1260326 and *TM6SF2* rs58542926 SNPs is significantly associated with a higher hepatic fat.^58^

Several other genes have been associated with hepatic fat accumulation and are currently under investigation in paediatric populations, including Lysophospholipase‐Like 1 (*LYPLAL1*) and Phosphatase 1 Regulatory Subunit 3B (*PPP1R3B*).^62^, ^63^ In addition, hepatic steatosis and fibrosis are individually heritable traits that share genetic influence with metabolic risk factors as insulin resistance, HDL, triglycerides and HbA1c,^64^, ^65^ and evidence from a family cohort study showed that first‐degree of patients with NAFLD‐cirrhosis have a higher risk of progressive NAFLD.^66^ Therefore, a better understanding of the role of genetics and family history in the pathogenesis and progression of youth‐onset NAFLD will drive prevention efforts in the future.

## 6 COMORBIDITIES IN CHILDREN WITH NAFLD

**FIGURE 1:**
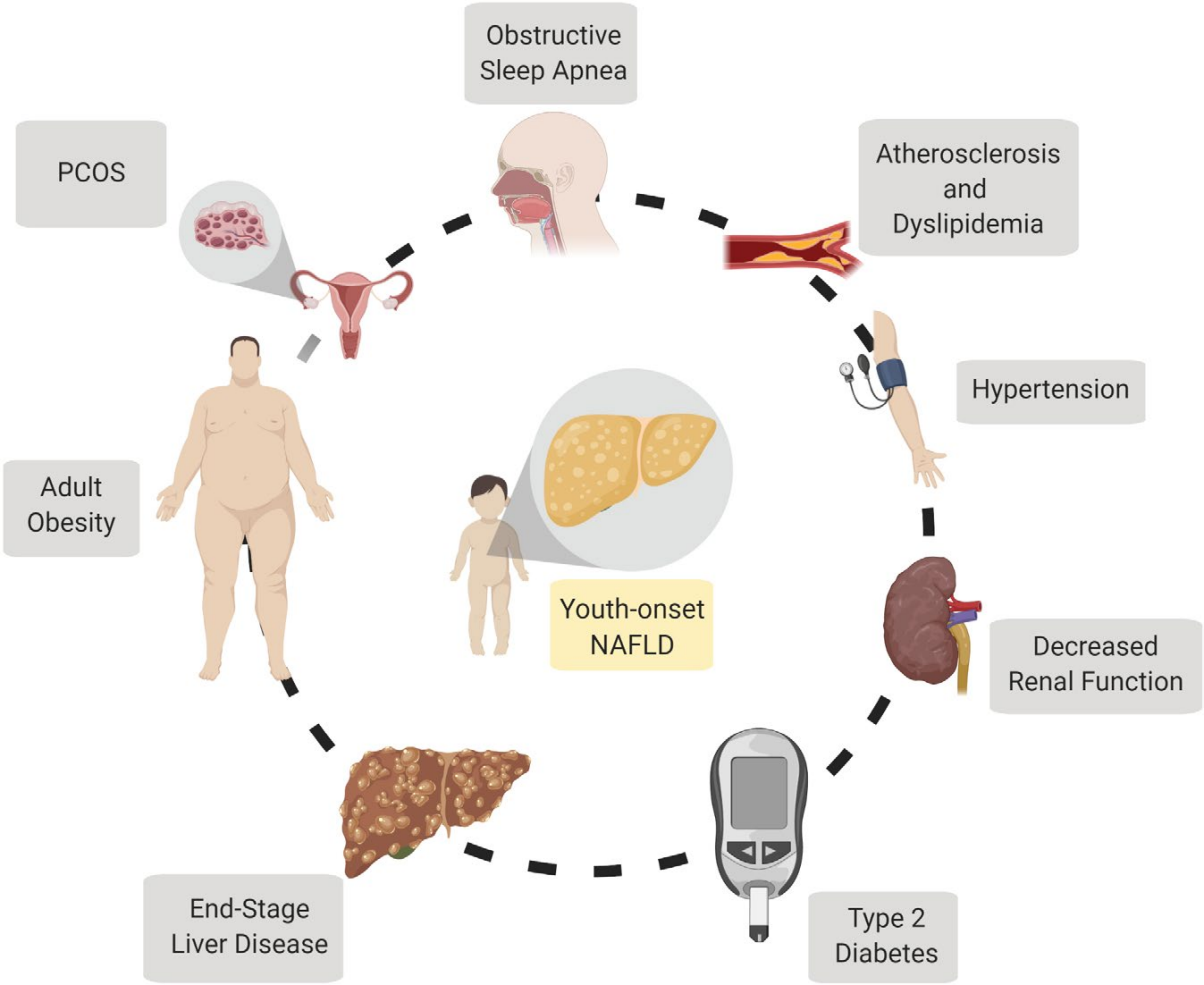
The whole child perspective. A summary of the potential health risks and comorbidities that may occur in children with youth‐onset NAFLD. NAFLD, Nonalcoholic fatty liver disease; PCOS, polycystic ovary syndrome

The health effects of youth‐onset NAFLD are not limited to the liver. Studies in children and adults have recognized NAFLD as a multiple‐organ condition (Figure 1). One of the most studied associations is between NAFLD and cardiovascular diseases (CVD). In the adult literature, the presence of NAFLD is a strong driver predictor of CVD events.^67^ In children, NAFLD is strongly associated with cardiovascular risk profiles, such as increased carotid intima‐media thickness, a surrogate marker of atherosclerosis,^68^, ^69^ a dyslipidemic profile characterized by high triglycerides, low‐density lipoprotein, and very‐low‐density lipoprotein,^70^ and high blood pressure.^71^ These associations not only support the presence of early‐onset of atherosclerosis, but also predict a greater burden of cardiovascular events in children with NAFLD. Likewise, a systematic review concluded that children with NAFLD, especially those with severe disease, are at risk for cardiac abnormalities such as left ventricular (LV) hypertrophy and diastolic LV dysfunction.^72^ NAFLD also increases the risk for high blood pressure, which, in a longitudinal study, was found to be present in 35.8% of the children with NAFLD.^71^

Multiple studies have explored the association between NAFLD and type 2 diabetes mellitus (T2DM), given several shared pathophysiological mechanisms including insulin resistance. Based on epidemiological studies, it appears as though the development of NAFLD precedes the development of T2DM, as evidenced by epidemiological studies showing that few children with NAFLD have T2DM, but the prevalence of abnormal glucose metabolism and/or T2D in children with NAFLD is extremely high (approximately 20%‐30%),^73^, ^74^ and by adulthood, more than half of patients with NAFLD have T2D.^75^ Moreover, children with NAFLD and type 2 diabetes were 3 times more likely to have NASH,^73^ suggesting the two pathologies may synergize to promote inflammation and exacerbate hepatic injury.

NAFLD has also been associated with decreased renal function in children and adults. Recently, in a study of 230 children with MRI‐diagnosed NAFLD, 21% presented with abnormal renal function (defined by an estimated glomerular filtration rate (eGFR) < 90 mL/min/1.73 m^2^, or microalbuminuria).^76^ Similarly, in another cohort of 179 children with biopsy‐proven NAFLD, glomerular hyperfiltration was presented in 20% and low eGFR in 15%.^77^ Although obesity duration and severity are also associated with a decreased renal function,^78^ NAFLD has been shown to be an independent predictor even after further adjustment for other metabolic risk factors and genetic polymorphisms like I148M (rs738409) of *PNPLA3*.^57^, ^79^

Other comorbidities that NAFLD have been associated with include obstructive sleep apnoea (OSA),^80^, ^81^ lower whole bone mineral density^82^ and polycystic ovary syndrome.^83^ In addition, youth‐onset NAFLD has been associated with a variety of psychosocial issues as low self‐esteem, psychological distress and poor quality of life.^84^ Thus, it is not surprising that, aligning with the evidence outlined above, the North American Society for Pediatric Gastroenterology, Hepatology and Nutrition (NASPGHAN) suggests screening for comorbidities such as T2DM, hypertension and dyslipidemia in children with NAFLD,^85^ and this list of comorbidities may increase in the future as more evidence is generated. However, despite these recommendations, only approximately half of paediatric gastroenterologists screen for those comorbidities.^86^ Acknowledgement and evaluation of NAFLD comorbidities is an essential tool that will reduce the impact of severe outcomes in young adults and is an important consideration for next‐generation, whole child treatment regimens.

## 7 SCREENING

**FIGURE 2:**
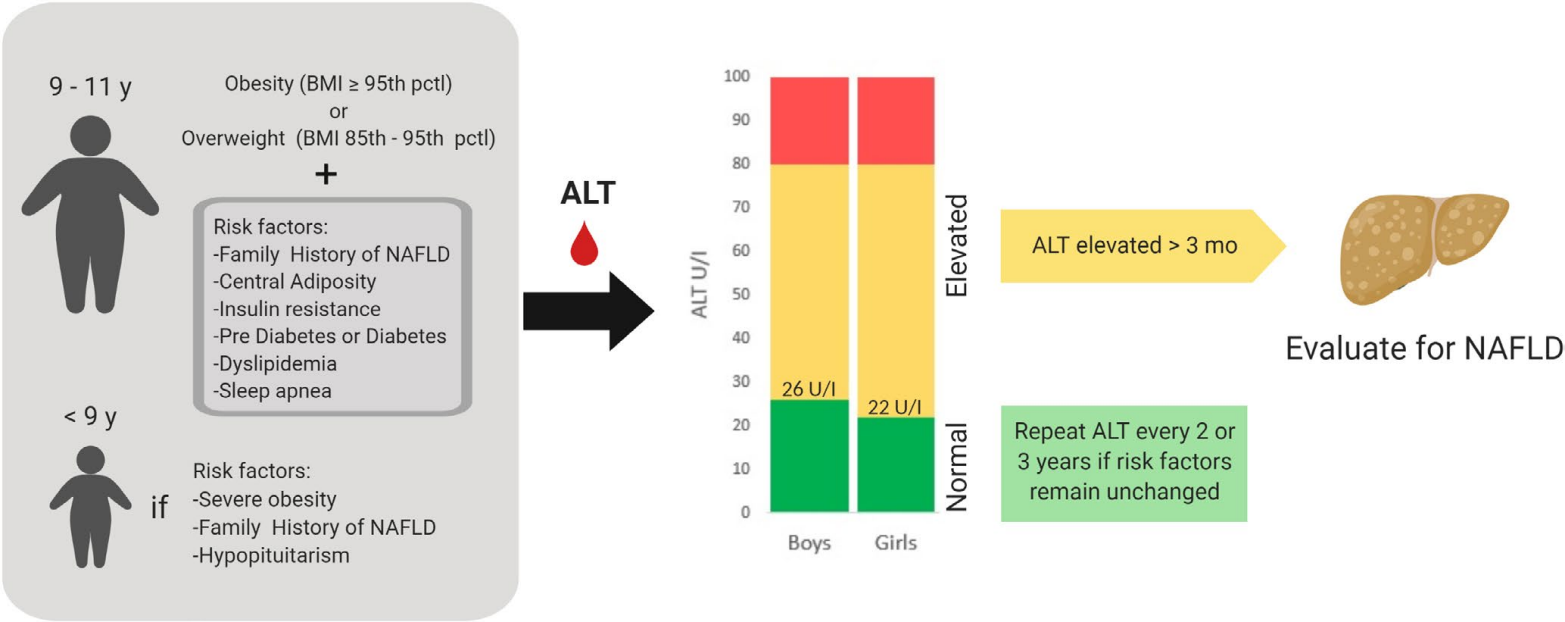
Schematic summary of the current screening guidelines for identifying children who may need further evaluation for youth‐onset NAFLD. ALT, alanine aminotransferase; NAFLD, Nonalcoholic fatty liver disease; y, years

Growing evidence of the hepatic and extra‐hepatic complications of NAFLD in children and young adults has emphasized the relevance of screening for NAFLD in the paediatric population. Recent NASPGHAN guidelines recommend screening children who are 9‐11 years old with obesity (BMI ≥ 95 percentile) or overweight (BMI ≥ 85 percentile) and other risk factors (such as insulin resistance, dyslipidemia, family history of NAFLD, sleep apnoea), as summarized in Figure 2.^85^ Specifically, based on the NASPGHAN guidelines, screening is currently based on ALT levels, and further evaluation is warranted if there is an ALT ≥ 80 U/L on initial screening, or if ALT is persistently elevated (>3 months) twice the upper limit of normal (in girls ALT ≥ 44 U/L, in boys ALT ≥ 52 U/L).^85^ However, it is important to note that the use of ALT for screening remains controversial and is not a universal recommendation. In addition, whether the above ALT cut‐points are the most appropriate for screening in children has been extensively discussed, and multiple efforts have been focused on redefining this limit. Two large population‐based studies that lay the groundwork for the NASPGHAN recommendations are the Canadian Laboratory Initiative in Pediatric Reference Intervals^87^ and the Screening ALT For Elevation in Today's Youth study.^88^ In the latter study, data from the National Health and Nutrition Examination Survey cycles 1999‐2006 were used to evaluate different ALT cut‐points for classifying NAFLD in children and found that a cut‐point of 25.6 U/L in boys and 22.1 U/L in girls achieved the highest sensitivity of 80% and 92%, and specificity of 79% and 85%, respectively.^88^ In another study of 408 children with NAFLD and obesity, ALT showed an accuracy of 80% for detecting NAFLD, based on slightly higher cut‐points of 42 U/L for boys (47.8% sensitivity, 93.2% specificity) and 30 U/L for girls (52.1% sensitivity, 88.8% specificity).^6^

The concomitant use of ultrasound for screening was also recommended by the European Society of Pediatric Gastroenterology, Hepatology and Nutrition (ESPGHAN) in 2012. Ultrasound indirectly estimates hepatic steatosis and remains one of the most widely used imaging modalities due to its cost and availability. However, in children it has low sensitivity and specificity, and based on the available evidence, a 2014 systematic review concluded that the use of ultrasound for NAFLD diagnosis or grading in youth is not recommended.^89^ In a study of children with obesity, with MRI as the reference ‘gold standard’ method, ALT and ultrasound sensitivity performed similarly in detecting NAFLD cases (44% vs 51%, respectively) and the concomitant use of both methods for screening didn't improve the diagnostic accuracy to detect NAFLD.^90^

Thus, overall, NAFLD screening in youth remains flawed, and the development of more accurate and precise noninvasive screening tests remains an active area of investigation, especially with regard to fibrosis, a key determinant of prognosis. To date, multiple scores for detecting and/or staging fibrosis have been proposed. The AST to platelet index ratio (APRI) and fibrosis‐4 index (FIB‐4) were developed in adults, but their performance on paediatric patients has been inconsistent, with areas under the receiving operating characteristic curve (AUROC) that range 0.67‐0.70 for APRI score, and 0.54‐0.81 for FIB‐4.^91^, ^92^ Other scores designed to predict fibrosis specifically in children include the Pediatric NAFLD Fibrosis Index (PNFI) and Pediatric NAFLD Fibrosis Score (PNFS). These scores achieved high AUC values in the development cohorts (0.74 for PNFS^93^ and 0.85 for PNFI^94^), but in validation studies the panels did not perform as well in predicting any stage of fibrosis with AUROC of 0.57 and 0.67, respectively.^95^ This may be because the validation cohort was predominantly Hispanic, while the development cohort was Caucasian. Other, more direct biomarkers of fibrosis such as Cytokeratin‐18, a marker of hepatocyte apoptosis, and plasma N‐terminal propeptide of type III procollagen (PIIINP), a marker of extracellular matrix remodelling, have shown promising results in children. In two paediatric studies, cytokeratin‐18 achieved moderate AUROCs ranging 0.66‐0.75 in classifying children with any stage of fibrosis vs none^96^, ^97^; whereas PIIINP was achieved an AUROC of 0.92 in classifying children with fibrosis stage 2 and up (F2, peri‐sinusoidal and portal).^98^

Metabolomics has also led to advancements in this field by allowing researchers to examine more systemic metabolic signatures that provide new insights to our understanding of processes involved in NAFLD. As an example, in a large paediatric study with 222 children with NAFLD (confirmed by liver biopsy or MRI) and 337 children without NAFLD, high‐resolution plasma metabolomics, measured by liquid chromatography mass spectrometry, was coupled with a multistep machine learning framework to select a screening panel of 11 metabolites features for classifying NAFLD vs. non‐NAFLD cases, which achieved a sensitivity of 73% and a specificity of 84%. With the addition of key clinical variables into the panel, which were also selected using machine learning, the specificity increased to 97%.^99^ While panels such as this one, as well as the other biomarkers describe above, require further validation, combination panels that incorporate established clinical assessments with novel biomarkers of disease have promise for improving the disease screening, diagnosis and monitoring in the future.

## 8 DIAGNOSIS

After screening, evaluation of a child with suspected NAFLD requires exclusion of alternative aetiologies such as viral hepatitis, drug‐induced fatty liver, Wilson disease, haemochromatosis, α‐1 antitrypsin deficiency, and autoimmune hepatitis. Once these diseases are excluded, we may suspect NAFLD and either a liver biopsy or imaging are needed to confirm the presence of hepatic steatosis. These two modalities will be discussed in detail next.

### 8.1 Liver pathology

To date, liver biopsy is required to assess the severity of inflammation and presence of nonalcoholic steatohepatitis (NASH), the more progressive subtype of NAFLD that is associated with fibrosis progression and poorer long‐term outcomes. Thus, NASPGHAN recommends performing a liver biopsy in children with risk of NASH and/or advanced fibrosis, considering risk factors such as ALT > 80 U/L, splenomegaly, AST/ALT > 1, type 2 diabetes, and panhypopituitarism. Likewise, in the context of fibrosis, a follow‐up liver biopsy is recommended over long time periods.^85^ In reality, most children with NAFLD do not undergo liver biopsy. However, when biopsied, approximately 1 in 4 children with NAFLD will have NASH or borderline NASH,^9^ and 15% have stage 3 fibrosis.^85^ In children, the presence of histological features of steatohepatitis are relevant in the evaluation of liver biopsy, but the location of steatosis and inflammation must be considered and may differ from the typical ‘adult’ form of NASH. Specifically, steatosis location in the periportal region (zone 1) is more common in younger children and has been associated with advanced fibrosis. Whereas, steatosis location at the centrilobular or pericentral region (zone 3) is more frequent in adolescents and has been associated with steatohepatitis.^100^ Similarly, hepatocyte ballooning is less frequent in the paediatric population, and inflammatory infiltrates arise more in the periportal region.^101^ Because of these differences, the classic definition of NASH seen in adults is often not met in children, even in the presence of substantial inflammation and fibrosis. These differences pose a challenge in the evaluation of youth‐onset NAFLD. Additional long‐term natural history studies of the various histologic patterns would be helpful for understanding the relevance of histological features to clinical outcomes.

### 8.2 Imaging

Imaging is a promising area for accurate quantification of hepatic steatosis and fibrosis in adults. However, fewer data are available in children. Controlled Attenuation Parameter (CAP), which is included in the transient elastography (TE) system of FibroScan®, achieved a high sensitivity and specificity (98.7% and 80%, respectively) in detecting the absence and presence of steatosis in children with NAFLD.^102^ However, CAP was limited in differentiating higher grades of steatosis in children with obesity, which is an important limitation given the strong link between obesity and NAFLD.^102^ In terms of fibrosis, TE achieved a moderate sensitivity and specificity (72% and 76%, respectively) for the detection of advanced fibrosis compared to minimal or no fibrosis in children with chronic liver diseases (37% with NAFLD).^103^ However, another paediatric study showed that the presence of liver inflammation might overestimate the liver stiffness measurement by TE in children with early fibrosis stages.^104^ One of the most sensitive techniques in children is magnetic resonance (MR)‐based techniques. Regarding hepatic steatosis quantification, MR‐spectroscopy‐proton density fat fraction (MRS‐PDFF) and MRI‐PDFF have shown to be very accurate and reliable methods in children.^105^, ^106^ Whereas, MR‐elastography, a technique designed to detect and stage liver fibrosis, showed a sensitivity of 88% and specificity of 85% for detecting advanced fibrosis in a cohort of children with chronic liver disease (77% of them had NAFLD). However, due to MR availability and costs, this method is mostly used in research.

## 9 MONITORING OF NAFLD SEVERITY

In clinical practice, paediatric NAFLD is monitored with serial measurements of ALT, ideally every 3‐6 months. Moreover, ALT is widely used as a surrogate marker of disease improvement in paediatric clinical trials. A secondary analysis of a multicenter randomized trial that included 178 children with biopsy‐proven NAFLD (TONIC trial) found that ALT was able to discriminate between those children that progressed from those that improved, based on a subsequent liver histology.^107^ Thus, although the single time‐point usage of ALT may not always directly reflect disease severity, changes in ALT levels over time appear to reflect the progression of the disease. This analysis was performed on a trial that only included children with ALT > 60 U/L; therefore, it is still uncertain whether ALT has a similar significance in those patients with low or normal ALT.

## 10 DISEASE MANAGEMENT

In the absence of pharmacological options in children, the mainstay recommendations for youth‐onset NAFLD therapy are focused on lifestyle changes, for example diet and exercise.^85^ In terms of diet, both ‘holistic’ dietary patterns and individual dietary components have been proposed for hepatic steatosis reduction and for the purpose of this narrative we will focus on the results from experimental studies thus far. The Mediterranean Diet (MD), which is rich in fruits and vegetables, olive oil, nuts/legumes, and fish, has been the most extensively tested dietary pattern and, in adults, there is consistent evidence that a MD treatment is associated with a reduction in hepatic fat^108^; The Dietary Approaches to Stop Hypertension (DASH) diet, which is rich in fruits and vegetables, low‐fat dairy, and unrefined grains, has also been tested in adults and shown to improve several biomarkers, including ALT levels (hepatic fat changes were not assessed).^109^ However, these diets have not been testing in children with NAFLD yet.

Dietary sugar is an individual dietary component that has been consistently implicated in NAFLD,^110^, ^111^ with evidence particularly supporting a mechanistic link between fructose and hepatic fat accumulation, as discussed in the ‘Pathogenesis’ section. Importantly, two recent intervention studies in children provide evidence to support the efficacy of dietary sugar restriction in NAFLD. The first study examined the effects of a short‐term (9‐day), dietary fructose restriction trial in 41 children (9‐18 years) and showed that the dietary treatment resulted in significant improvement not only in hepatic fat, but also in DNL rates and certain insulin kinetics.^112^ The second study was a longer‐term (8‐week), randomized controlled dietary feeding trial in 40 adolescent boys (11‐16 years) with NAFLD that tested the effect of a very low free sugar diet (<3% of total energy intake) on hepatic fat and several secondary outcomes.^113^ Similarly, this study showed that the dietary sugar restriction was associated with a significant improvement in liver steatosis (25% over 8 weeks), independent of total weight loss, providing strong evidence for the potential benefits of sugar reduction in youth‐onset NAFLD.

Dietary fat may be another target for managing NAFLD,^114^ and overall, it appears that hypocaloric, low‐fat diets have been shown to induce a similar improvement in hepatic fat compared to low‐carbohydrate/low‐sugar diets.^115^ For example, in a small (n = 17) pilot study of children with NAFLD that compared the effects of a low‐fat diet vs a low‐glycaemic diet for 6 months, both diets were associated with a similar improvement in liver fat (−8.8% in the low‐glycaemic diet vs. −10.5% in the low‐fat diet), as well as ALT levels.^116^ What remains a controversy is whether isocaloric changes in dietary fat or dietary carbohydrates, for example without changes in total energy intake or weight, are more effective for NAFLD management and well‐controlled studies are needed to examine this. In addition, studies with larger and more generalizable samples and with longer‐term durations are needed to confirm the effectiveness and sustainability of these dietary changes.

Nutrigenomics is another emerging area of research that may be applicable for NAFLD management by considering the interaction between diet and genetics.^117^ An interesting example is from a study by Davis et al that found that, in a cohort of 153 Hispanic children, hepatic fat correlated with carbohydrate and sugar intake only among those children with the PNPLA3 GG genotype, but not those with the CC or CG genotype.^118^ This suggests there may be significant inter‐individual variation in the response to certain dietary interventions, which are driven by underlying genetics, and this should be leveraged to design more personalized interventions.

In addition to dietary modifications, multidisciplinary weight management programs that include coordinated treatment/counselling with a psychologist, exercise physiologist, registered dietitian, medical provider, and nurse provide higher success rate and long‐lasting lifestyle modifications that could benefit children with NAFLD.^119^ There is also continual interest in pharmacological and surgical options for paediatric NAFLD. As reviewed elsewhere, examples of potential pharmacological treatments of interest include insulin sensitizers, antioxidants (especially vitamin E), lipid‐lowering medications, bile acids and probiotics.^120^ Importantly, the majority of pharmacological intervention trials have been limited to adults, and thus far surgical intervention studies focused specifically on outcomes in obese children and adolescents with NAFLD are lacking.^121^ It is imperative that there are also independent paediatric trials that are designed with certain considerations in mind, such as the different histological subtypes in children, and the potential confounding by age, sex and pubertal stage.^122^

## 11 TRANSITIONING FROM YOUTH‐ONSET NAFLD TO ADULT NAFLD

The dramatic increase of youth‐onset NAFLD^123^ has prompted attention to the transition of NAFLD patients to adult care. Currently, studies are lacking that describe the natural history of paediatric NAFLD into young adulthood. There are, however, cross‐sectional studies that support a rise in NAFLD among young adults. In the US the estimated prevalence of NAFLD doubled in participants 18‐35 years, from 9.6% in 1988‐1994 to 24% in 2005‐2010^124^—a trend that paralleled prevalence rates in adolescence.^123^ This is concerning given the health risks of NAFLD, especially its potential to progress to end‐stage liver disease, which is underscored by the findings of a study by Alkhouri et al showing progressive increases in the frequency of NASH as the primary indication for liver transplant from 2001‐2012 among young adults (<40 years old).^125^

Future studies examining this transition period must also consider the challenges that may be faced by patients during this time. This transition is multifactorial and not only involves the patient, but also involves the family and health care providers. From a psychosocial perspective, some patients will join the workforce, others may start college, others may be doing both, while most of them will move away from their childhood home. This has an impact on their lifestyle choices, including eating behaviours and physical activity, which previously may have been supported by their parents. As they age into adulthood, other factors also come into play including potential increases in substance abuse and high‐risk sexual behaviour and consequently sexually transmitted diseases (such as hepatitis B or C).^126^ Young adults are also more likely to be uninsured due to entry‐level jobs and uncertain finances.^127^ Thus, because of these social factors, and because NAFLD is a mostly asymptomatic disease without effective pharmacotherapy, there is high likelihood that patients with youth‐onset NAFLD will be lost to follow‐up during their 20's.

In order to adequately address the transition to adult care, planning has to begin during early adolescence, taking into account the educational, emotional, and psychosocial needs of the patient. The main goal is to promote the patient's development into an independent young adult, who has an active role in self‐care activities. These activities involve building long‐lasting healthy dietary and physical activity habits. In order to achieve that, patient education is an important precursor of readiness. Moreover, nonadherence is higher among older adolescents and young adults; therefore, patient‐centred education should be emphasized to ensure full understanding of the benefits of treatment and medical follow‐up.^128^, ^129^ Likewise, sufficient communication between the paediatric and adult medical team is essential. Taken together, the transition to adult care is a multifactorial and dynamic process that warrants more research, but, if done satisfactorily, might prevent future comorbidities and costs in patients with NAFLD.

## 12 CONCLUDING REMARKS

In summary, youth‐onset NAFLD increases the risk of morbidity and mortality lifelong. While our understanding of the pathophysiology, diagnosis and treatment of NAFLD in childhood and adolescence is growing, methods to prevent NAFLD in children have not yet been studied. Treatment strategies should focus on sugar restriction and the adoption of other beneficial dietary behaviours, along with sufficient exercise. The severity of youth‐onset NAFLD is best assessed by liver biopsy at this time, although the long‐term outcome(s) of various histologic patterns is not yet known. Transition into adulthood is a high‐risk time period for youth with NAFLD. While much is known, the biggest gaps in knowledge stem from the lack of long‐term natural history data, and future research will be needed to understand the outcomes of youth‐onset NAFLD and the best methods for preventing and/or slowing the high prevalence rates.

## CONFLICT OF INTEREST

MV has consulting arrangements with Boehringer Ingelheim, Bristol Myers Squibb, Intercept, Mallinckrodt, Novo Nordisk and TARGET PharmaSolutions and research funding from TARGET PharmaSolutions and Bristol Myers Squibb.

## AUTHORS CONTRIBUTIONS

EC‐L and MBV were responsible for the conception of the manuscript and figures, and writing the original draft. CEC assisted with writing, reviewing and editing the final manuscript.

## Data Availability

Data sharing was not applicable to this article as no data sets were generated or analysed during the current study.
